# An intra-bacterial activity for a T3SS effector

**DOI:** 10.1038/s41598-020-58062-y

**Published:** 2020-01-23

**Authors:** Samir El Qaidi, Nichollas E. Scott, Michael P. Hays, Brian V. Geisbrecht, Shelby Watkins, Philip R. Hardwidge

**Affiliations:** 1 College of Veterinary Medicine, Kansas State University, Manhattan, KS 66506 USA; 2 Department of Microbiology and Immunology, University of Melbourne within the Peter Doherty Institute for Infection and Immunity, Melbourne, 3000 Australia; 3 Department of Biochemistry and Molecular Biophysics, Kansas State University, Manhattan, KS 66506 USA

**Keywords:** Bacterial pathogenesis, Bacterial physiology

## Abstract

Many Gram-negative bacterial pathogens interact with mammalian cells by using type III secretion systems (T3SS) to inject virulence proteins into host cells. A subset of these injected protein ‘effectors’ are enzymes that inhibit the function of host proteins by catalyzing the addition of unusual post-translational modifications. The *E. coli* and *Citrobacter rodentium* NleB effectors, as well as the *Salmonella enterica* SseK effectors are glycosyltransferases that modify host protein substrates with N-acetyl glucosamine (GlcNAc) on arginine residues. This post-translational modification disrupts the normal functioning of host immune response proteins. T3SS effectors are thought to be inactive within the bacterium and fold into their active conformations after they are injected, due to the activity of chaperones that keep the effectors in a structural state permissive for secretion. While performing mass spectrometry experiments to identify glycosylation substrates of NleB orthologs, we unexpectedly observed that the bacterial glutathione synthetase (GshB) is glycosylated by NleB on arginine residue R256. NleB-mediated glycosylation of GshB resulted in enhanced GshB activity, leading to an increase in glutathione production, and promoted *C. rodentium* survival in oxidative stress conditions. These data represent, to our knowledge, the first intra-bacterial activity for a T3SS effector and show that arginine-GlcNAcylation, once thought to be restricted to host cell compartments, also plays an important role in regulating bacterial physiology.

## Introduction

The NleB (*Escherichia coli* and *Citrobacter rodentium*) and SseK (*Salmonella enterica*) enzymes are type three secretion system (T3SS) effector proteins that glycosylate host proteins with N-acetyl glucosamine (GlcNAc) on arginine residues to subvert their function in the innate immune system^1–3^. Arginine glycosylation is unusual because it occurs on the guanidinium groups of arginines, which are poor nucleophiles. The NleB/SseK orthologs share high degrees of structural similarity and consist of a catalytic domain including essential DXD and HEN motifs, a helix-loop-helix (HLH) domain, and a C-terminal lid domain^4–6^. Several ‘death domain’-containing proteins such as the Fas-Associated protein with Death Domain (FADD), tumor necrosis factor receptor type 1-associated death domain protein (TRADD), and the receptor interaction serine/threonine-protein kinase 1 (RIPK1) are NleB/SseK substrates^2^. These effectors disrupt tumor necrosis factor receptor (TNFR)-associated factor (TRAF) signaling, leading to inhibition of the pro-inflammatory NF-κB pathway^1–3^.

T3SS effectors are chaperoned in the bacterium after their synthesis to keep them partially unfolded and competent for secretion, as well as for targeting the effectors to the T3SS sorting platform^7^. The chaperones are then stripped from their effector substrates at the sorting platform and the effectors are secreted in an unfolded conformation^8^. T3SS effectors are generally believed to be inactive until they are injected into host cells, where they then fold into their active conformations^9^.

Some precedent for type IV secretion system (T4SS) effector activity in both the bacterium and the host may exist in plant pathogens. *Agrobacterium tumefaciens* transfers a nucleoprotein complex into plant cells. The VirD2 protein is associated with the transferred DNA (T-DNA). VirD2 has endonuclease activity within the bacterium to initiate T-DNA transfer^10^. VirD2 also targets the nucleoprotein complex in the plant cell nucleus, where it assists in integrating T-DNA into plant chromosomes^11^. Therefore, VirD2 may have enzymatic functions both within the bacterium and in the host plant cell. A recent study also indicated that *Yersinia pseudotuberculosis* uses the secreted T3SS translocator YopD to control RNA regulators and increase the abundance of LcrF, a common transcriptional activator of other T3SS effector genes^12^.

N-linked protein glycosylation on arginine has also been reported for the EarP glycosyltransferase from *E. coli* and *Pseudomonas aeruginosa*^13^. In this case, a single arginine rhamnosylation event activates the function of the polyproline-specific bacterial translation elongation factor EF-P^13^. Although the relatively inert guanidine group of arginine was previously thought to impede nucleophilic attack onto donor substrates, it is now clear that many bacterial enzymes have overcome this barrier. The enzymology and functional roles of arginine glycosylation, methylation, phosphorylation, and ADP-ribosylation were recently reviewed^14^.

While characterizing additional NleB glycosylation substrates, we made the unexpected observation that the bacterial glutathione synthetase (GshB) is glycosylated by NleB on an arginine residue. This glycosylation contributes to bacterial survival in hydrogen peroxide stress conditions by enhancing GshB activity and increasing intracellular levels of glutathione (GSH). Thus, NleB is active and performs important biological functions within the bacterium, prior to its secretion.

## Results

### NleB glycosylates GshB

We performed mass spectrometry experiments to identify new glycosylation substrates of NleB orthologs from EHEC strains associated with human disease outbreaks. These experiments were conducted by infecting HEK293T cells with EHEC strains that express NleB1. We then used an anti-Arg-R-GlcNAc monoclonal antibody^15^ for the antibody-based capture of arginine-GlcNAcylated peptides to perform proteome-wide assessment of Arg-GlcNAcylation mediated by NleB1, as described previously^16^. We unexpectedly observed glycosylation of *E. coli* glutathione synthetase (GshB) on arginine residue R256 as the most abundant Arg-GlcNAcylated peptide enriched from samples, followed by the known human NleB1 target FADD^16^ (Supp. Table 1).

**Figure 1:**
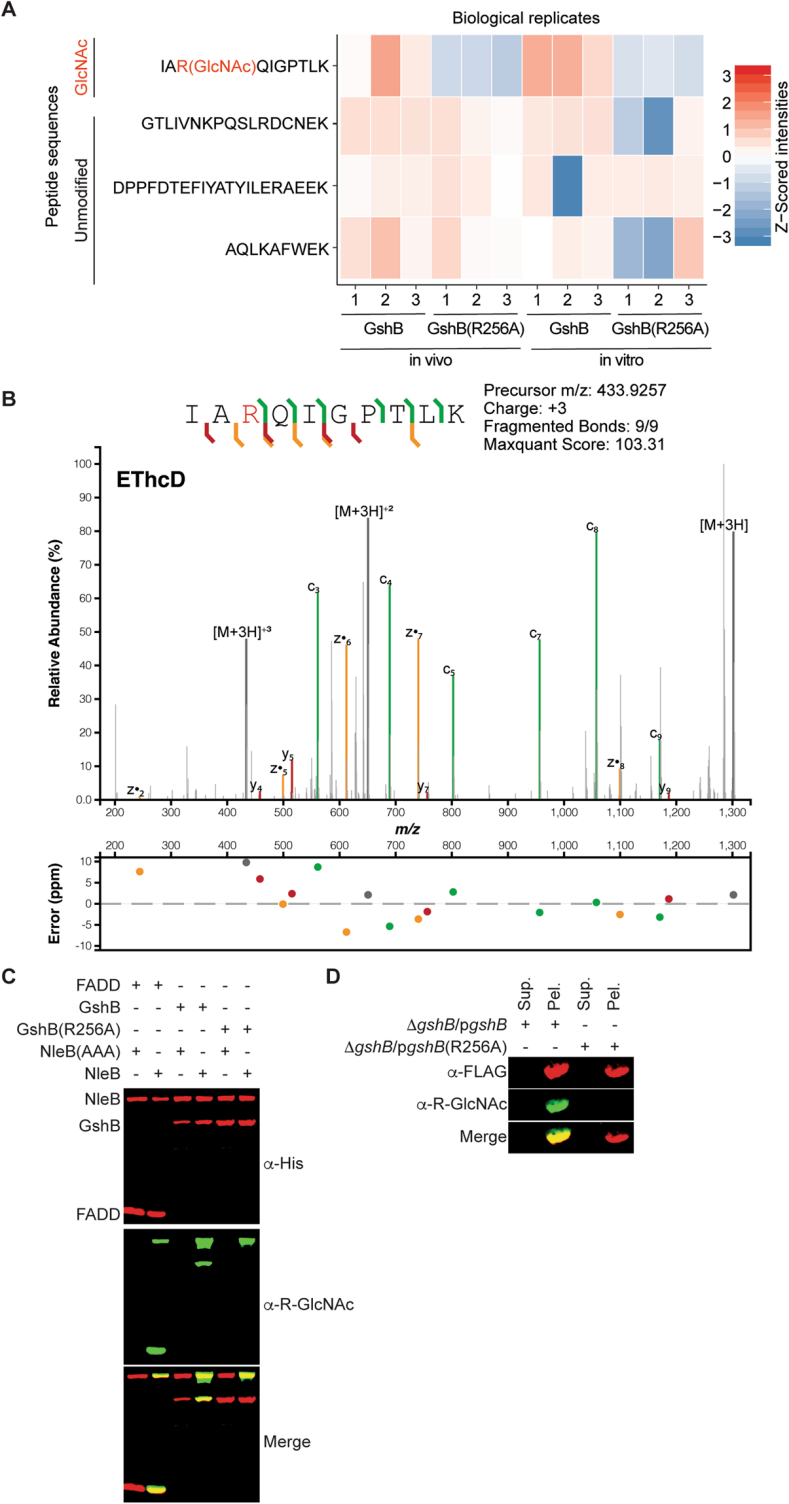
NleB glycosylates GshB R256. (**A**) Heatmap of Z-scored ion intensities of GshB peptides demonstrates that glycosylated R256 is observed only within WT GshB samples in both *in vivo* and *in vitro* glycosylation assays. The abundance of non-glycosylated peptides ^109^GTLIVNKPQSLRDCNEK^125^, ^88^DPPFDTEFIYATYILERAEEK^108^ and ^145^AQLKAFWEK^153^ are unaltered across assays (**B**) EThcD spectra of the *in vivo* glycosylated GshB peptide ^254^IARQIGPTLK^263^ confirms glycosylation is localized to R256. (**C**) *Western* blot analysis of *in vitro* GshB glycosylation assays. (**D**) *Western* blot analysis of *in vivo* GshB glycosylation assays; Sup, culture supernatant; Pel, bacterial lysate.

Because *C. rodentium* encodes only one copy of NleB, while most EHEC strains encode two copies (NleB1 and NleB2), we subsequently attempted to reproduce our initial findings using *C. rodentium* NleB and GshB using *in vivo*^4^ or *in vitro*^17^ studies to investigate glycosylation within this protein. Using these assays, we observed that GshB is exclusively modified on R256, with this glycosylation event absent in GshB(256 A) expressing strains [Fig. 1A; -log10(p-value) of 1.77 and 2.78 from *in vivo* and *in vitro* assays respectively, Supp. Table 2]. To further test the localization of the glycosylation, *in vivo* glycosylated GshB was subjected to Electron-Transfer/Higher-Energy Collision Dissociation (EThcD) fragmentation, confirming the attachment of the GlcNAc residue to R256 (Fig. 1B). These data support the notion that the glutathione synthetase GshB from the attaching/effacing pathogens EHEC and *C. rodentium* is glycosylated at R256 under *in vivo* conditions.

To corroborate our mass spectrometry data, we conducted *in vitro* glycosylation assays^17^ with the Anti-R-GlcNAc monoclonal antibody^15^ by expressing recombinant forms of wild-type (WT) GshB or GshB(256 A). NleB glycosylated the WT, but not the R256A GshB mutant (Fig. 1C), consistent with our *in vivo* and *in vitro* MS assays. Within these assays, FADD was used as a positive control as a known NleB substrate^16^. The NleB(AAA) mutant, which lacks glycosylation activity^1^, was used as a negative control.

We then generated a *gshB* deletion in *C. rodentium* and complemented this mutant with FLAG-tagged versions of either WT or GshB(R256A). Both FLAG-tagged forms of GshB were isolated from *C. rodentium*; only WT GshB was glycosylated by the endogenous levels of NleB (Fig. 1D). Neither secretion of GshB nor its glycosylation in the culture medium by NleB was observed, suggesting that NleB glycosylation of GshB occurred within the bacterium.

### NleB promotes oxidative stress resistance

Glutathione (GSH) is generated in Gram-negative bacteria in a two-step process by γ-glutamylcysteine synthetase (*gshA*) and glutathione synthetase (*gshB*). GshA uses glutamate and cysteine as substrates to catalyze the formation of γ-L-glutamylcysteine. GshB catalyzes GSH production by ligating glycine and γ-L-glutamylcysteine^18^. *Salmonella* lacking either enzyme do not produce GSH and exhibit increased susceptibility to oxidative stress^19^. A *Salmonella gshA* deletion is hypersensitive to H_2_O_2_, and *gshA* and *gshB* deletions are hypersensitive to both nitric oxide (NO) and S-nitrosoglutathione (GSNO)^19^. Similarly, in *Pseudomonas aeruginosa*, *gshA* and/or *gshB* deletions are more sensitive to environmental stress and attenuated for virulence^20^. In *Streptococcus pneumoniae*, mutating *gshT*, an ABC transporter required for GSH import, increased *S. pneumoniae* sensitivity to superoxide, and was attenuated in a mouse model of infection^21^. Deleting *gshA* and *gshB* from *P. aeruginosa* attenuates several virulence-associated phenotypes including motility and biofilm formation. GSH was also shown to activate both the *P. aeruginosa* T3SS and a subset of T6SS genes^22^. Glutathione binding to the *Listeria monocytogenes* master regulator PrfA is also critical to the virulence of this intracellular pathogen^23^.

**Figure 2:**
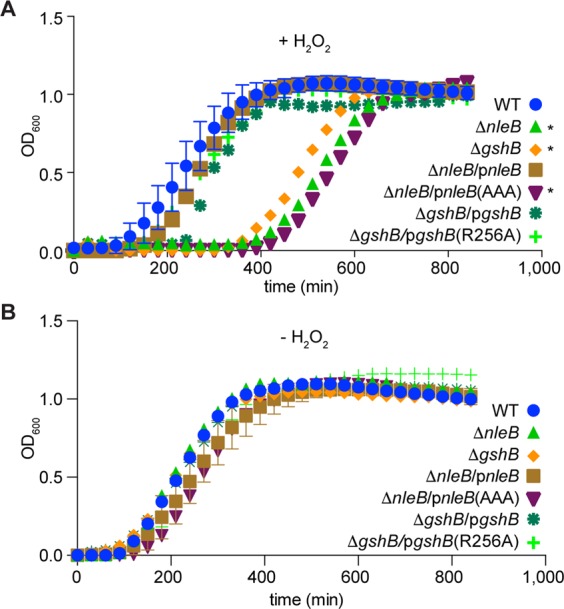
*C. rodentium* growth assays. (**A**) Quantification of *C. rodentium* growth (OD_600_) as a function of time (min) in the presence of 2.4 mM H_2_O_2_. (**B**) *C. rodentium* growth in the absence of H_2_O_2_.

To determine whether GshB glycosylation by NleB has functional significance in promoting resistance to oxidative stress, we compared the growth rates of bacterial strains in the presence or absence of H_2_O_2_. Both the *nleB* and *gshB* mutants had a significant growth defect in the presence of H_2_O_2_ (Fig. 2A), despite having similar growth rates in the absence of H_2_O_2_ (Fig. 2B). The *nleB* mutant phenotype was complemented by expressing WT *nleB* on a plasmid, but not by expressing the inactive *nleB*(AAA) mutant. Mutating the GshB R256 residue to alanine (R256A) had no impact on bacterial growth rates. These data highlight a new role for NleB, namely its requirement for *C. rodentium* survival in peroxide stress conditions.

### Arg-glycosylation enhances GshB activity

**Figure 3:**
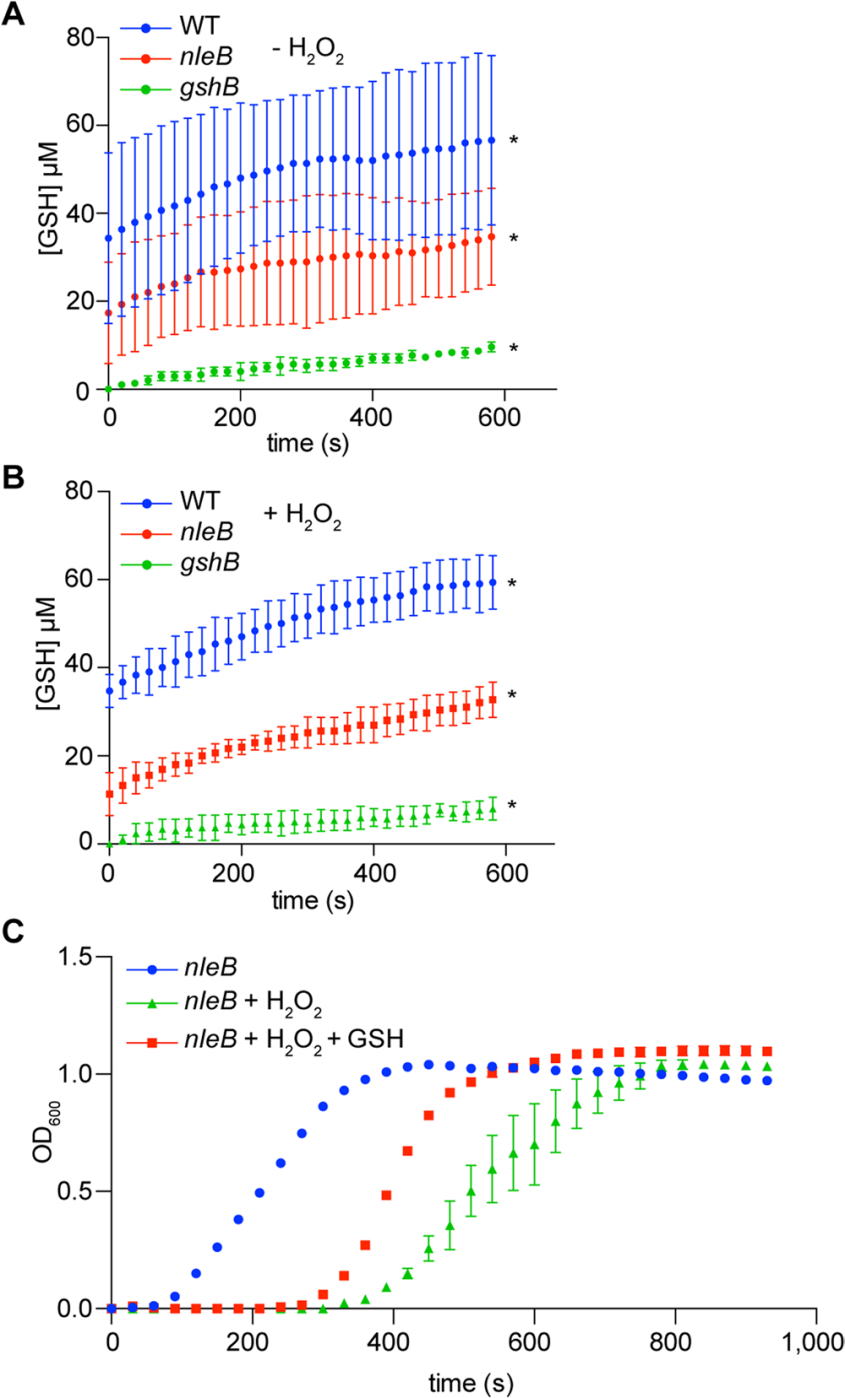
GSH production from bacterial lysates. (**A**) Bacterial lysates derived from *C. rodentium* strains grown in the absence of H_2_O_2_ were incubated with 1 mM CDNB and 1 µM GST. Absorbance was recorded at 340 nm every 20 seconds for 10 minutes and OD_340_ data were converted into GSH concentrations using a GST standard curve. (**B**) GSH production from *C. rodentium* strains grown in the presence of H_2_O_2_. (**C**) Complementation of *C. rodentium nleB* growth in 2.4 mM H_2_O_2_ in the presence or absence of 5.0 mM GSH.

We then tested the hypothesis that GshB glycosylation enhances GshB-mediated production of glutathione (GSH), consistent with the bacterial growth phenotypes in the presence of H_2_O_2_. We incubated *C. rodentium* lysates prepared from cultures grown in the presence or absence of 2.4 mM H_2_O_2_ with glutathione *S*-transferase (GST) and 1-chloro-2,4-dinitrobenzene (CDNB) to quantify GSH concentrations in bacterial lysates^24^. The WT strain produced significantly more GSH (3.4 + 0.9 pmoles/s) than did the *nleB* mutant (2.3 + 0.6 pmoles/s), irrespective of the presence of H_2_O_2_ (Fig. 3A,B). To verify that the growth defect of the *nleB*-deleted strain in H_2_O_2_ was due to insufficient GSH, we supplemented bacterial cultures with 5.0 mM GSH and observed partial restoration of the growth of the *nleB* mutant in the presence of H_2_O_2_ (Fig. 3C).

**Figure 4:**
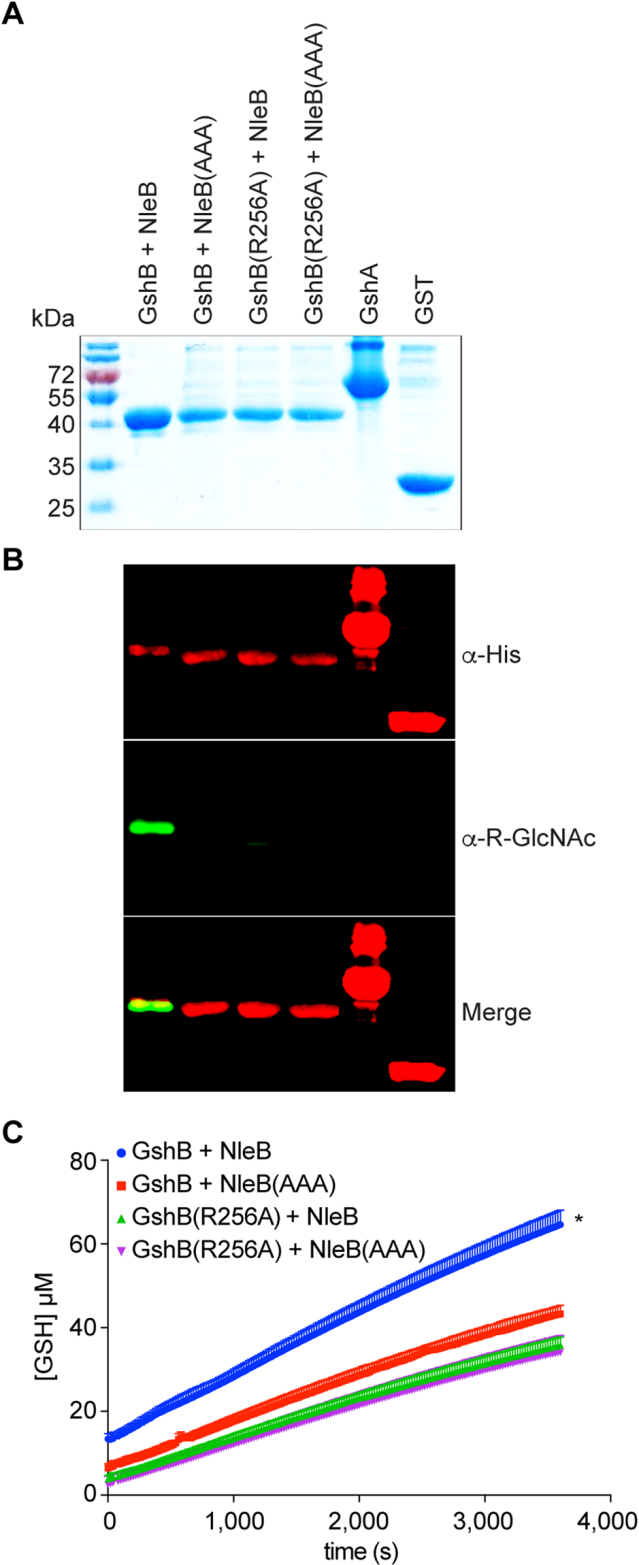
*In vitro* GSH assays. (**A**) Coomassie blue staining of purified proteins used in GSH assays. (**B**) Western blot analysis of the GshB glycosylation state from GSH assays. (**C**) GSH production as a function of time from reactions containing either WT or GshB(256A) purified after their co-expression with either WT or NleB(AAA).

We then reconstituted an *in vitro* GSH production assay using purified, recombinant GshA, GST, as well as GshB that was purified from *E. coli* BL21(DE3), following its co-expression with either NleB or NleB(AAA) (Fig. 4A). Thus, we aimed to generate either glycosylated GshB (when co-expressed with WT NleB) or unglycosylated GshB [when mutated to R256A and/or when WT GshB was co-expressed with NleB(AAA)] to directly characterize the impact of GshB glycosylation on GshB activity. Glycosylation of GshB on R256 in this assay was confirmed using western blotting, showing that NleB was active when expressed in *E. coli* BL21(DE3) (Fig. 4B). The GshB enzyme purified from the WT NleB co-expression strain was significantly more active than the GshB enzyme purified from the NleB(AAA) co-expression strain (1.5 + 0.1 vs. 1.0. + 0.1 pmoles/s) (Fig. 4C). NleB had no impact on GSH production catalyzed by GshB(R256A). We obtained similar data when GshB was first purified from *E. coli* and then subsequently glycosylated by NleB *in vitro* (Fig. S1).

We also considered whether *Salmonella* might glycosylate GshB *in vivo*. We did not observe such glycosylation under conditions performed similarly to those using *C. rodentium* (Fig. S2A). However, *Salmonella* is significantly more resistant to H_2_O_2_ than is *C. rodentium*, due to the presence of redundant catalases and alkyl hydroperoxide reductases^25–27^. Consistent with this, we failed to observe a growth phenotype when we subjected WT *Salmonella* and all possible combinations of *sseK1/K2/K3* mutants to 12.0 mM H_2_O_2_, a concentration 5-times greater than that used for *C. rodentium* experiments (Fig. S2B). These data suggest that the SseK enzymes are not significantly involved in H_2_O_2_ resistance by *Salmonella*.

However, we further considered the possibility that some degree of GshB glycosylation might be observed *in vitro* using purified recombinant proteins. We especially considered this because of previous findings demonstrating that NleB/SseK substrate specificity may be more broad *in vitro* as compared to *in vivo* assays, particularly when the enzyme is present at supraphysiological concentration^16^. We incubated GshB with all known NleB/SseK orthologs from EHEC, EPEC, *C. rodentium*, and *Salmonella* and observed that EHEC NleB1, EPEC NleB1, and *Salmonella* SseK1 all glycosylated GshB, whereas NleB2, SseK2, and SseK3 did not (Fig. S2C). Whether GshB glycosylation by EHEC and/or EPEC NleB1 may affect oxidative stress resistance in these attaching/effacing pathogens awaits further experimentation.

Although no chaperone for NleB has been identified, we also considered whether the presence of CesT, a multi-cargo chaperone that interacts with at least 9 other effectors^28^, might affect NleB activity. We especially considered this possibility because effector-binding and secretion activities are separable for CesT^29^. However, we did not observe any impact on NleB activity in a *C. rodentium cesT* mutant (Fig. S2A), nor did we observe an interaction between NleB and CesT, as assessed using affinity chromatography (*data not shown*). Thus, NleB activity appears to be independent of CesT.

## Discussion

NleB is an arginine glycosyltransferase that modifies several host proteins, leading to inactivation of the host pro-inflammatory response mediated by NF-κB^4,6^. Here we discovered that NleB also plays a significant role in bacterial survival in oxidative stress conditions by glycosylating GshB to enhance GSH production. In this case, rather than inactivating the NleB substrates, as is seen with the mammalian targets, Arg-GlcNAcylation significantly activates the bacterial GshB enzyme. It remains to be determined why GshB glycosylation enhances its enzyme activity. The glycosylated R256 is distant from the active site (Fig. S2B), as inferred from the *E. coli* GshB structure [PDB 1GSA;^30^]. Possible mechanisms could include changes in GshB oligomerization state, a conformational change of the GshB active site, or enhanced affinity for/localization with GshA.

GshB is an abundant cytoplasmic protein^31^. In our assays, we observed a 50–100% increase in GshB activity (Figs. 3–4**)**, despite only a relatively low degree of overall GshB Arg-GlcNAcylation (occupancy of ~5%, Supp. Table 1). It is therefore possible that Arg-GlcNAcylation may increase GshB activity by as much as 10-fold. Formal testing of this hypothesis awaits the complete separation of glycosylated from unglycosylated forms of GshB.

NleB/SseK activities in the host cell have been characterized for their inhibition of proteins involved in the innate immune response^1–4,6^. Previous work with Ear-P showed that Arg-rhamnosylation activates EF-P in the bacterial cytosol^13^, to avoid ribosome stalling during the synthesis of poly-proline regions^32^. It is now clear from our work that Arg-GlcNAcylation also provides an important activating function, namely the NleB-mediated activation of GshB through R256 glycosylation. The abundance and functional significance of cytoplasmic bacterial glycoproteins may be a fruitful area for future studies.

Regardless of the specific activating mechanism, these data represent, to our knowledge, the first example of a T3SS effector functioning within both the bacterium and within the host cell. It is possible that other *C. rodentium* proteins are glycosylated by NleB to provide the organism a means by which to integrate T3SS activation and effector synthesis with bacterial physiological processes such as transcriptional regulation, flagellar biosynthesis, and quorum sensing. It is also possible that other T3SS effectors are substrates of NleB, and that other T3SS effectors with enzymatic activities are active within the bacterium, a concept similar to the role of metaeffectors playing important regulatory roles in *Legionella pneumophila* biology^33^. The extent to which other effectors with enzymatic activities may be active within the bacterium may emerge as a new area of investigation.

## Materials and Methods

### Strains and molecular cloning

The plasmids and strains used in this study are listed in Table 1. WT *nleB* (*C. rodentium*) and its derivative DAD^221–223^/AAA were cloned into pET42a. FADD was cloned into pET15a. WT *gshB* and its derivative *gshB* R256A, as well as *gshA*, were cloned in pET28a using ABC cloning^34^. The *C. rodentium gshB* deletion was generated using lambda red recombination^35^.

**Table 1:** Plasmids and strains used in this study.

Plasmid	Source
FLAG-NleB (*C. rodentium*)	^1^
FLAG-NleB (*C. rodentium*) (DAD^221–223^/AAA)	^1^
GST-NleB (*C. rodentium*)	^17^
GST-NleB (*C. rodentium*) (DAD^221–223^/AAA)	^17^
GST-NleB1 (EHEC)	^17^
GST-NleB1 (EPEC)	^17^
GST-NleB2	^17^
GST-SseK1 (*S. enterica*)	^17^
GST-SseK2 (*S. enterica*)	^17^
GST-SseK3 (*S. enterica*)	^17^
His-FADD	^17^
His-GshA	This study
His-GshB	This study
His-GshB(R256A)	This study
FLAG-His-GshB	This study
FLAG-His-GshB(R256A)	This study
His-CesT	This study
His-GST	Novagen
**Strain**	**Source**
*C. rodentium* DBS100	^44^
*C. rodentium* DBS100 Δ*nleB*	^1^
*C. rodentium* DBS100 Δ*nleB*/pFLAG-CTC-*nleB*	^1^
*C. rodentium* DBS100 Δ*nleB*/pFLAG-CTC-*nleB* DAD^221–223^/AAA	^1^
*C. rodentium* DBS100 Δ*cesT*	^45^
*C. rodentium* Δ*gshB*	This study
*C. rodentium* Δ*gshB*/pFLAG-CTC *gshB*	This study
*C. rodentium* Δ*gshB*/pFLAG-CTC *gshB*(R256A)	This study
*S. enterica*	^46^
*S. enterica ΔsseK1*	^46^
*S. enterica ΔsseK2*	^46^
*S. enterica ΔsseK3*	^46^
*S. enterica ΔsseK1ΔsseK2*	^46^
*S. enterica ΔsseK1ΔsseK3*	^46^
*S. enterica ΔsseK2ΔsseK3*	^46^
*S. enterica ΔsseK1ΔsseK2ΔsseK3*	^46^
*E. coli* BL21(DE3) x pET28a-*gshA*	This study
*E. coli* BL21(DE3) x pET28a-*gshB*	This study
*E. coli* BL21(DE3) x pET28a-*gshB*(R256A)	This study
*E. coli* BL21(DE3) x pET42a-*nleB*	This study
*E. coli* BL21(DE3) x pET42a-*nleB* DAD221^-223^/AAA	This study
*E. coli* BL21(DE3) x pET42a-*nleB1* (EHEC)	^17^
*E. coli* BL21(DE3) x pET42a-*nleB1* (EPEC)	^17^
*E. coli* BL21(DE3) x pET42a-*nleB2*	^17^
*E. coli* BL21(DE3) x pET42a-*sseK1*	^17^
*E. coli* BL21(DE3) x pET42a-*sseK2*	^17^
*E. coli* BL21(DE3) x pET42a-*sseK3*	^17^
*E. coli* O111:NM R82F2	^36^

### Protein purification

Proteins were expressed from *E. coli* BL21 (DE3) and induced with 0.5 mM IPTG when cultures reached an OD_600_ of 0.4. Cells were grown for an additional 4 h at 37 °C and then harvested using centrifugation. Cell pellets were resuspended in 1/40^th^ culture volume of 50 mM NaH_2_PO_4_ pH 8.0 supplemented with 0.5 mg/ml lysozyme and protease inhibitor cocktails (Thermo Scientific). Bacterial suspensions were incubated on ice for 30 min with occasional shaking, at which time an equal volume 50 mM NaH_2_PO_4_ pH 8.0, 2 M NaCl, 8 mM imidazole, 20% glycerol, 2% Triton X-100 was added for additional incubation on ice for 30 min. Cell lysates were sonicated, centrifuged, and combined with 2 ml Ni-NTA slurry (Qiagen) for 1 h at 4 °C with gentle rotation. The mixture was loaded on a Poly-Prep Chromatography Column (Bio-Rad) and washed in 50 mM NaH_2_PO_4_ pH 8.0, 600 mM NaCl, 60 mM imidazole, 10% glycerol). Proteins were eluted in 50 mM NaH_2_PO_4_ pH 8.0, 600 mM NaCl, 250 mM imidazole, 10% glycerol and then dialyzed into the same buffer lacking imidazole^17^.

### Enrichment of arginine-glycosylated peptides from cell lysates

HEK293T cells were grown in DMEM to 80% confluency and then infected with EHEC O111:NM R82F2^36^ at a multiplicity of infection of 1.0 for 16 h. Infected cells were washed three times in ice-cold PBS and lysed by scraping with ice-cold guanidinium chloride lysis buffer (6 M GdmCl, 100 mM Tris pH 8.5, 10 mM TCEP, 40 mM 2-Chloroacetamide) on a bed of ice. Lysates were collected and boiled at 95 °C for 10 minutes with shaking at 2,000 rpm to shear DNA and inactivate protease activity. Lysates were then cooled for 10 minutes on ice and then boiled again at 95 °C for 10 minutes with shaking at 2,000 rpm. Protein samples (2 mg) were acetone precipitated and dried protein pellets were resuspended in 6 M urea, 2 M thiourea, 40 mM NH_4_HCO_3_ and reduced/alkylated prior to digestion with Lys-C (1/200 w/w) and then with trypsin (1/50 w/w) overnight as previously described^37^. Digested samples were acidified to a final concentration of 0.5% formic acid and desalted with 50 mg tC18 SEP-PAK (Waters Corporation). tC18 SEP-PAKs were conditioned with buffer B (80% ACN, 0.1% formic acid), washed with 10 volumes of Buffer A* (0.1% TFA, 2% ACN), sample loaded, column washed with 10 volumes of Buffer A* and bound peptides eluted with buffer B then dried.

Arg-GlcNAc peptide affinity purification was performed as previously described^16^. Protein A/G plus Agarose beads (Santa Cruz, Santa Cruz CA) were washed with immunoprecipitation buffer (IAP, 10 mM Na_2_HPO_4_, 50 mm NaCl, 50 mM MOPS, pH 7.2) and rotated overnight with 10 μg of anti-Arg-GlcNAc antibody (ab195033, Abcam) at 4 °C. Coupled anti-Arg-GlcNAc beads were then washed with 100 mM sodium borate (pH 9) to remove non-bound proteins and cross-linked for 30 minutes of rotation using 20 mM dimethyl pimelimidate in 100 mM HEPES, pH 8.0. Cross-linking was quenched by washing beads three times with 200 mM ethanolamine, pH 8.0 and then rotating the beads in an additional 1 ml of 200 mM ethanolamine, pH 8.0 for 2 hours at 4 °C. Purified peptides were resuspended in 1 ml IAP buffer and peptide lysates were then added to the prepared cross-linked anti-Arg-GlcNAc antibody beads and rotated for 3 hours at 4 °C. Antibody beads were centrifuged at 3,000 g for 2 minutes at 4 °C and the unbound peptide lysates collected. Antibody beads were then washed with ice-cold IAP buffer and Arg-GlcNAc peptides eluted using two rounds of acid elution. For each elution round, 100 μl of 0.2% TFA was added and antibody beads allowed to stand at room temperature with gentle shaking every minute for 10 minutes. Peptide supernatants were collected and desalted using C18 stage tips^38^ before being dried down and stored until LC-MS analysis.

### SP3 on-bead Lys-C digestion of purified GshB

Purified tagged GshB was cleaned up using SP3 based purification according to previous protocols^39^. Samples were first denatured and reduced using 1% SDS, 10 mM DTT, 100 mM HEPES by boiling at 95 °C, 1,000 rpm for 10 minutes. Samples were then cooled and alkylated with 40 mM 2-chloroacetamide (CAA) for 1 hour at RT in the dark. The alkylation reactions were then quenched with 40 mM DTT for 10 minutes and then samples precipitated on to SeraMag Speed Beads (GE Healthcare, USA) with ethanol (final concentration 50% v/v). Samples were shaken for 10 minutes to allow complete precipitation onto beads and then washed three times with 80% ethanol. The beads were resuspended in 100 mM ammonium bicarbonate containing 1 μg lys-C 1/50 (w/w) and digested overnight at 37 °C. Samples were centrifuged at 14,000 g for 5 minutes to pellet the beads and the supernatant collected and desalted using C18 stage tips before being dried for LC-MS analysis.

### Identification of arginine-glycosylated affinity enriched peptides and his-tagged proteins using reversed phase LC-MS

Purified peptides were resuspended in Buffer A* and separated using a two-column chromatography setup composed of a PepMap100 C18 20 mm × 75 μm trap and a PepMap C18 500 mm × 75 μm analytical column (Thermo Fisher Scientific)^16^. Samples were concentrated onto the trap column at 5 μl/min for 5 minutes and infused into either an Orbitrap Fusion Lumos Tribrid Mass Spectrometer (Thermo Fisher Scientific) for the analysis of enriched Arg-GlcNAc peptides and PRM analysis of Arg-GlcNAcylated GshB or an Orbitrap Elite (Thermo Fisher Scientific) for the comparison of Arg-GlcNAcylation levels within purified GshB. Gradients (120 minutes) were run by altering the buffer composition from 1% buffer B to 28% B over 90 minutes, then from 28% B to 40% B over 10 minutes, then from 40% B to 100% B over 2 minutes, the composition was held at 100% B for 3 minutes, and then dropped to 3% B over 5 minutes and held at 3% B for another 10 minutes for enriched Arg-GlcNAc modified peptides and comparison of Arg-GlcNAcylation levels within purified GshB. For 120-minute gradients, the Lumos and Elite Mass Spectrometers were operated in a data-dependent mode automatically switching between the acquisition of a single Orbitrap MS scan HCD or CID fragmentation. For parallel reaction monitoring (PRM) experiments, the known Lys-C Arg-modified peptide of GshB (IARQIGPTLKEK) was monitored using EThcD fragmentation targeting the predicted m/z for the +2 (m/z 650.2835) and +3 charge (m/z 488.9248) states over a 95 minute gradient, altering the buffer composition from 1% buffer B to 28% B over 60 minutes, then from 28% B to 40% B over 10 minutes, then from 40% B to 100% B over 2 minutes, held at 100% B for 3 minutes, and then dropped to 3% B over 5 minutes and held at 3% B for another 15 minutes.

### Mass spectrometry data analysis

Identification of Arg-glycosylated peptides was accomplished using MaxQuant (v1.5.3.30)^40^. Searches were performed against *E. coli* O127:H6 strain E2348/69 (Uniprot proteome id UP000008205- *E. coli* O127:H6 strain E2348/69/EPEC, downloaded 28-07-2014, 4,595 entries) or *C. rodentium* ICC168 (Uniprot proteome id UP000001889- *C. rodentium* strain ICC168 downloaded 12/12/2016) proteomes depending on the samples, with carbamidomethylation of cysteine set as a fixed modification. Searches were performed with Trypsin or Lys-C cleavage specificity depending on the experiment. Two miscleavage events were allowed, as well as the variable modifications of oxidation of methionine, N-Acetylhexosamine addition to arginine (Arg-GlcNAc) and acetylation of protein N-termini. Precursor mass tolerance was set to 20 parts-per-million (ppm) for the first search and 10 ppm for the main search, with a maximum false discovery rate (FDR) of 1.0% set for protein and peptide identifications. The Match Between Runs option was enabled with a precursor match window set to 2 minutes and an alignment window of 10 minutes. For label-free quantitation, the MaxLFQ option within Maxquant^41^ was enabled, in addition to the re-quantification module. Peptide outputs were processed using the Perseus (v1.4.0.6)^42^ analysis environment to remove reverse matches and common protein contaminants with missing values imputed and the peptide intensities z-scored. MS/MS annotations were undertaken using the Interactive Peptide Spectral Annotator^43^. Mass spectrometry proteomics data were deposited to the ProteomeXchange Consortium via the PRIDE partner repository with the dataset identifier PXD015752.

### Glycosylation assays

*In vitro* glycosylation assays were performed as described previously^17^ using 200 nM of NleB1 or its orthologs with 1 μM of either WT GshB or GshB R256A in 50 mM Tris-HCl pH 7.4, 1 mM UDP-GlcNAc, 10 mM MnCl_2_, and 1 mM DTT. After 2 h incubation at RT, samples were subjected to western blotting using an anti-R-GlcNAc monoclonal antibody (Abcam). *In vivo* glycosylation assays were performed using *C. rodentium* and *S. enterica* strains electroporated with plasmids expressing His-GshB or His-GshB(R256A). Transformed bacteria were grown overnight in the presence of 0.5 mM IPTG, harvested using centrifugation, and then His-tagged proteins were purified as described above, and then subjected to western blotting using an anti-R-GlcNAc monoclonal antibody (Abcam).

### Bacterial growth assays

Overnight cultures were used to inoculate 50 ml of LB medium. H_2_O_2_ (2.4 mM for *C. rodentium* or 12.0 mM for *S. enterica*) was added to cultures when they reached an OD_600_ of 0.3 and bacterial growth was monitored for 10–16 h at 37 °C.

### GSH quantification from bacterial lysates

Bacteria were harvested by centrifugation, lysed in 50 mM Tris-HCl pH 7.4 supplemented with 0.5 ug/ml lysozyme, and recentrifuged. The supernatant was incubated with 1 mM CDNB and 1 µM GST. Absorbance was recorded at 340 nm every 20 seconds for 10 minutes and OD_340_ data were converted into molar concentrations of GSH using a GST standard curve^24^.

### *In-vitro* GSH assays

His-GshB or His-GshB(R256A) were purified over Ni-NTA slurries after they were respectively co-expressed with either NleB-FLAG or NleB(AAA)-FLAG in *E. coli* BL21(DE3) cells. An *in vitro* GSH assay was then prepared by first incubating GshA (1 µM) with 5 mM glycine, 5 mM cysteine, and 5 mM glutamate in a reaction buffer containing 50 mM Tris HCl pH 7.4, 1 mM DTT, 1 mM MgCl_2_, 1 mM ATP, and 3% DMSO. After 1 h incubation at 37 °C, the purified GshB proteins (50 nM), along with 100 nM GST and 1 mM CDNB were added, and glutathione formation was monitored by reading the absorbance at 340 nm as a function of time.

### Statistical analyses

Bacterial growth assays were analyzed using non-linear regression followed by Dunn’s multiple comparison testing. GSH assays were analyzed using linear regression. p-values <0.05 were considered significant.

## Supplementary information

Supporting Information.

Supporting Information2.

Supporting Information3.

Supporting Information4.

Supporting Information5.

Supporting Information6.
