# Supplementary material for: An intra-bacterial activity for a T3SS effector
Source: Sci Rep. 2020 Jan 23;10:1073. doi: 10.1038/s41598-020-58062-y (PMC6978387; doi:10.1038/s41598-020-58062-y)
Supplement: Supplementary file 1 — Supporting Information. [file 41598_2020_58062_MOESM1_ESM.pdf]

Intensity 1 (Sample type A)	Intensity 2 (Sample type A)	Intensity 12 (Sample type A)	Intensity 4 (Sample type B)	Intensity 5 (Sample type B)	Intensity 6 (Sample type B)	Intensity 7 (Sample type C)	Intensity 8 (Sample type C)	Intensity 10 (Sample type C)
26.9453	28.7039	26.3549	28.2234	30.5265	31.0709	24.6823	26.0919	23.9121
27.9434	30.1422	26.4111	28.5587	26.0365	25.9343	25.3852	26.9125	26.5347
34.0575	34.2248	34.2331	34.4048	34.2786	33.9295	34.3379	31.1804	34.2507
23.4929	17.7389	34.3247	34.0517	34.5522	34.8677	33.9609	34.0582	34.0816
23.5449	17.8237	26.6242	30.2371	30.7279	30.0911	27.1545	28.0322	28.5849
29.6897	27.5836	31.439	34.6382	33.2036	33.139	31.6066	31.2172	31.219
19.3057	16.7454	17.2452	25.308	26.3319	25.8922	18.486	17.5812	18.9957
34.767	34.7574	34.358	34.6698	33.0203	32.5514	33.8871	34.3863	34.4993
27.1132	30.5305	18.7918	28.6181	26.7057	29.2988	18.8861	16.783	24.8916
35.8465	35.6867	35.2241	35.8726	35.2381	36.0202	35.2584	35.02	35.149
32.9659	33.7568	31.2424	33.981	32.158	34.4368	32.5529	31.1666	32.5034
26.0953	31.5174	29.143	27.5854	22.5883	26.3956	35.6773	36.0507	30.635
22.1544	28.8411	23.4635	17.148	16.9465	15.4692	28.1941	28.0473	24.7248
22.0485	30.5096	18.2548	17.8751	16.5361	17.1327	17.6605	17.5033	17.8212
35.0504	34.7219	34.6232	35.4329	35.9848	35.0333	35.3013	34.6851	34.4134
29.8145	30.3875	26.8741	30.8629	30.418	32.2557	26.1423	26.8394	27.8365
25.1848	24.413	22.9823	17.3615	26.6252	26.0785	25.2043	24.345	26.4285
33.8399	34.2206	34.0422	33.2052	33.3952	33.2315	27.813	34.1811	33.8147
29.0653	30.7076	28.961	28.3337	29.7795	29.9786	29.2182	29.3771	29.0204
34.5534	34.898	34.6646	34.8205	35.4921	35.0988	34.6682	34.6692	34.096
33.6947	34.2284	32.1771	34.239	34.189	34.3418	32.5607	32.2417	32.6232

Intensity 9 (Sample type D)	Intensity 11 (Sample type D)	Intensity 3 (Sample type D)	C: Modifications	C: Raw file
27.1483	16.6008	30.9456	Unmodified	Nsco_20190925_Pulldowns_PH_B3_lysC
17.4587	15.5167	29.5803	Unmodified	Nsco_20190925_Pulldowns_PH_A3_lysC
34.2721	34.2987	34.2054	Unmodified	Nsco_20190925_Pulldowns_PH_D3_lysC
34.1648	22.5635	17.6977	Unmodified	Nsco_20190925_Pulldowns_PH_D3_lysC
27.6654	16.4007	19.4128	Oxidation (M)	Nsco_20190925_Pulldowns_PH_C1_lysC
31.1844	17.5629	17.8867	Unmodified	Nsco_20190925_Pulldowns_PH_B1_lysC
16.9213	17.1782	16.8674	Oxidation (M)	Nsco_20190925_Pulldowns_PH_B1_lysC
25.6865	17.7472	33.0871	Unmodified	Nsco_20190925_Pulldowns_PH_A2_lysC
24.0694	17.335	29.9886	Unmodified	Nsco_20190925_Pulldowns_PH_B3_lysC
35.2202	28.575	35.6333	Unmodified	Nsco_20190925_Pulldowns_PH_B3_lysC
31.0498	25.9559	34.5179	Oxidation (M)	Nsco_20190925_Pulldowns_PH_A2_lysC
28.5149	15.6578	34.1036	Unmodified	Nsco_20190925_Pulldowns_PH_C1_lysC
18.0175	18.7334	17.6827	Arg_GlcNAc	Nsco_20190925_Pulldowns_PH_C1_lysC
16.699	17.5832	24.3707	Unmodified	Nsco_20190925_Pulldowns_PH_A2_lysC
35.3985	30.3039	36.3193	Unmodified	Nsco_20190925_Pulldowns_PH_A1_lysC
26.8286	17.2696	30.9709	Unmodified	Nsco_20190925_Pulldowns_PH_B3_lysC
16.8004	16.3006	29.5456	Unmodified	Nsco_20190925_Pulldowns_PH_A3_lysC
33.9102	25.2006	32.9303	Unmodified	Nsco_20190925_Pulldowns_PH_D3_lysC
29.0019	18.4756	29.6034	Oxidation (M)	Nsco_20190925_Pulldowns_PH_C3_lysC
34.9892	26.7941	35.7217	Unmodified	Nsco_20190925_Pulldowns_PH_C1_lysC
32.146	23.6202	33.8039	Oxidation (M)	Nsco_20190925_Pulldowns_PH_B3_lysC

C: Student's T-test Significant A_BA_vs_B	C: Student's T-test significant	C: Student's T-test Significant C_DC_vs_D	C: Student's T-test significant _	N: Mass	N: Mass Fractional Part	N: Retention time	N: Calibrated retention time	N: PEP
				2914.55	0.552754	53.433	54.114	5.60E-147
				1119.61	0.60768	37.401	38.598	3.65E-35
				2546.22	0.221686	73.826	74.063	1.48E-100
				3460.68	0.675525	66.106	66.61	5.29E-74
+	A_B			3476.67	0.670439	56.769	57.27	1.02E-24
+	A_B			3875.85	0.845837	56.933	57.499	7.22E-13
+	A_B			3891.84	0.840752	55.366	55.353	1.15E-21
				1971.02	0.020759	36.786	37.912	2.31E-145
				4191.18	0.178663	57.881	58.217	1.52E-66
				2253.23	0.230358	53.839	54.12	0
				2269.23	0.225272	49.824	50.265	3.83E-135
				1095.68	0.676428	45.359	46.31	1.87E-11
+	A_B	+	C_D	1298.76	0.755801	27.767	29.229	0.005964
				1352.81	0.813984	25.663	26.255	1.99E-11
				2238.17	0.168469	60.65	60.753	0
				2678.44	0.443187	58.67	59.157	5.96E-87
				3339.77	0.765584	62.232	62.907	6.92E-16
+	A_B			1509.86	0.858885	67.22	67.598	4.83E-153
				1525.85	0.8538	46.442	46.851	3.25E-95
				1637.95	0.953848	56.84	56.938	3.51E-143
				1653.95	0.948763	47.62	47.902	9.06E-206

N: Score	N: Delta score	N: Intensity	N: MS/MS Count	N: -Log Student's T-test p-value A_BA_vs_B	N: Student's T-test Difference A_BA_vs_B	N: Student's T-test Test statistic A_BA_vs_B
187.04	177.09	7.1E+09	38	1.09166	-2.60555	-2.32185
166.07	145.23	3.13E+09	23	0.405911	1.32241	0.95708
175.77	169.35	2.24E+11	321	0.0744764	-0.0324961	-0.212084
148.26	142.25	1.68E+11	102	0.891021	-9.30507	-1.91151
97.049	93.187	5.34E+09	41	1.38714	-7.68774	-2.97336
74.581	70.124	6.08E+10	45	1.54802	-4.0895	-3.3597
96.913	87.962	1.88E+08	7	3.18923	-8.07858	-9.6431
215.93	199.62	1.94E+11	237	0.859616	1.21361	1.84893
132.48	123.45	3.99E+09	18	0.312161	-2.72907	-0.764184
304.91	289.7	5.38E+11	1027	0.15303	-0.12453	-0.409691
205	191.83	1.12E+11	440	0.355878	-0.870246	-0.855122
119.68	100.59	1.51E+11	402	0.713009	3.39544	1.5606
96.342	60.021	1.11E+09	5	1.76577	8.29843	3.92685
133.88	111.24	1.55E+09	8	0.816308	6.42297	1.76319
300.18	281.77	4.65E+11	607	1.05712	-0.685192	-2.24969
164.69	160.42	1.36E+10	33	0.816615	-2.15352	-1.76379
92.551	83.787	1.18E+09	13	0.0978422	0.838336	0.273117
206.46	206.46	1.44E+11	207	2.42535	0.756914	6.05473
206.32	181.19	8.38E+09	100	0.100128	0.214022	0.278974
210.14	204.87	3.64E+11	596	0.91798	-0.431782	-1.96553
228.93	228.26	1.38E+11	353	0.65337	-0.88987	-1.44435

N: -Log Student's T-test p-value C_DC_vs_D	N: Student's T-test Difference C_DC_vs_D	N: Student's T-test Test statistic C_DC_vs_D	T: Sequence	T: Proteins
0.000207792	-0.00276693	-0.000637792	AFWEKHSDIILKPLDGMGGASIFRVK	sp|P04425
0.541662	5.42557	1.22641	AQLKAFWEK	sp|P04425
0.409891	-1.00239	-0.96511	DPPFDTEFIYATYILERAEEK	sp|P04425
0.879536	9.22487	1.88858	EGDPNLGVIAETLTEHGTRYCMAQNYLPAI	sp|P04425
0.931999	6.76424	1.99374	EGDPNLGVIAETLTEHGTRYCMAQNYLPAI	sp|P04425
0.952472	9.13625	2.03509	EGDPNLGVIAETLTEHGTRYCMAQNYLPAI	sp|P04425
-4.34E-08	0	0	EGDPNLGVIAETLTEHGTRYCMAQNYLPAI	sp|P04425
0.922146	8.75061	1.9739	GTLIVNKPQSLRDCNEK	sp|P04425
0.340172	-3.61074	-0.822675	GTLIVNKPQSLRDCNEKLFTAWFSDLTPET	sp|P04425
0.365041	1.99964	0.873948	HSDIILKPLDGMGGASIFRVK	sp|P04425
0.244898	1.56643	0.619742	HSDIILKPLDGMGGASIFRVK	sp|P04425
0.630794	8.02888	1.40038	IARQIGPTLK	sp|P04425
2.77866	8.84421	7.53099	IARQIGPTLK	sp|P04425
0.319253	-1.88933	-0.779075	IARQIGPTLKEK	sp|P04425
0.157201	0.792708	0.419643	LFTAWFSDLTPETLVTRNK	sp|P04425
0.178141	1.9164	0.468944	LFTAWFSDLTPETLVTRNKAQLK	sp|P04425
0.434967	4.44376	1.01547	LFTAWFSDLTPETLVTRNKAQLKAFWEK	sp|P04425
0.134487	1.2559	0.364857	LGIVMDPIANINIK	sp|P04425
0.413058	3.5116	0.971491	LGIVMDPIANINIK	sp|P04425
0.276805	1.97614	0.689042	LGIVMDPIANINIKK	sp|P04425
0.343436	2.61851	0.829438	LGIVMDPIANINIKK	sp|P04425

T: Gene Names	T: Protein Names	T: Identification type 1	T: Identification type 2	T: Identification type 3	T: Identification type 4	T: Identification type 5	T: Identification type 6	T: Identification type 7
gshB	Glutathion	By MS/MS	By MS/MS	By MS/MS	By MS/MS	By MS/MS	By MS/MS	By MS/MS
gshB	Glutathion	By MS/MS	By MS/MS	By MS/MS	By MS/MS	By MS/MS	By MS/MS	By matchin
gshB	Glutathion	By MS/MS	By MS/MS	By MS/MS	By MS/MS	By MS/MS	By MS/MS	By MS/MS
gshB	Glutathion	By MS/MS			By MS/MS	By MS/MS	By MS/MS	By MS/MS
gshB	Glutathion	By MS/MS			By MS/MS	By MS/MS	By MS/MS	By MS/MS
gshB	Glutathion	By MS/MS	By matching		By MS/MS	By MS/MS	By MS/MS	By MS/MS
gshB	Glutathione synthetase				By MS/MS	By MS/MS	By MS/MS	
gshB	Glutathion	By MS/MS	By MS/MS	By MS/MS	By MS/MS	By MS/MS	By MS/MS	By MS/MS
gshB	Glutathion	By MS/MS	By MS/MS	By MS/MS	By MS/MS	By MS/MS	By MS/MS	
gshB	Glutathion	By MS/MS	By MS/MS	By MS/MS	By MS/MS	By MS/MS	By MS/MS	By MS/MS
gshB	Glutathion	By MS/MS	By MS/MS	By MS/MS	By MS/MS	By MS/MS	By MS/MS	By MS/MS
gshB	Glutathion	By MS/MS	By MS/MS	By MS/MS	By MS/MS	By MS/MS	By MS/MS	By MS/MS
gshB	Glutathion	By matchin	By MS/MS					By MS/MS
gshB	Glutathion	By MS/MS	By MS/MS	By MS/MS				
gshB	Glutathion	By MS/MS	By MS/MS	By MS/MS	By MS/MS	By MS/MS	By MS/MS	By MS/MS
gshB	Glutathion	By MS/MS	By MS/MS	By MS/MS	By MS/MS	By MS/MS	By MS/MS	By MS/MS
gshB	Glutathion	By MS/MS	By MS/MS	By MS/MS		By matchin	By matchin	By MS/MS
gshB	Glutathion	By MS/MS	By MS/MS	By MS/MS	By MS/MS	By MS/MS	By MS/MS	By MS/MS
gshB	Glutathion	By MS/MS	By MS/MS	By MS/MS	By MS/MS	By MS/MS	By MS/MS	By MS/MS
gshB	Glutathion	By MS/MS	By MS/MS	By MS/MS	By MS/MS	By MS/MS	By MS/MS	By MS/MS
gshB	Glutathion	By MS/MS	By MS/MS	By MS/MS	By MS/MS	By MS/MS	By MS/MS	By MS/MS

T: Identification type 8	T: Identification type 9	T: Identification type 10	T: Identification type 11	T: Identification type 12	M: Protein group IDs
By MS/MS	By MS/MS	By MS/MS		By MS/MS	869;843
By MS/MS		By MS/MS		By MS/MS	869;843
By MS/MS	By MS/MS	By MS/MS	By MS/MS	By MS/MS	869;843
By MS/MS	By MS/MS	By MS/MS	By MS/MS	By MS/MS	869;843
By MS/MS	By MS/MS	By MS/MS		By MS/MS	869;843
By MS/MS	By MS/MS	By MS/MS		By MS/MS	869;843
					869;843
By MS/MS	By MS/MS	By MS/MS		By MS/MS	869;843
	By MS/MS	By MS/MS			869;843
By MS/MS	By MS/MS	By MS/MS	By MS/MS	By MS/MS	869;843
By MS/MS	By MS/MS	By MS/MS	By MS/MS	By MS/MS	869;843
By MS/MS	By MS/MS	By MS/MS	By matchin	By MS/MS	869
By MS/MS		By matching		By MS/MS	869
					869
By MS/MS	By MS/MS	By MS/MS	By MS/MS	By MS/MS	869;843
By MS/MS	By MS/MS	By MS/MS		By MS/MS	869;843
By MS/MS		By MS/MS		By MS/MS	869;843
By MS/MS	By MS/MS	By MS/MS	By MS/MS	By MS/MS	869;843
By MS/MS	By MS/MS	By MS/MS		By MS/MS	869;843
By MS/MS	By MS/MS	By MS/MS	By MS/MS	By MS/MS	869;843
By MS/MS	By MS/MS	By MS/MS	By MS/MS	By MS/MS	869;843

Intensity 1 (Sample type A)	Intensity 2 (Sample type A)	Intensity 12 (Sample type A)	Intensity 4 (Sample type B)	Intensity 5 (Sample type B)	Intensity 6 (Sample type B)	Intensity 7 (Sample type C)	Intensity 8 (Sample type C)	Intensity 10 (Sample type C)
0.044956	0.48887	-0.10406	0.367583	0.948925	1.08635	-0.52625	-0.17044	-0.72067
0.535939	1.02515	0.195022	0.67284	0.111679	0.088942	-0.03323	0.306579	0.222531
0.09543	0.28368	0.293039	0.486349	0.344345	-0.04879	0.411073	-3.14379	0.312864
-0.87313	-1.69181	0.668024	0.629187	0.700401	0.74529	0.616258	0.630107	0.63343
-0.39383	-1.53175	0.218642	0.937224	1.03486	0.908186	0.324115	0.49868	0.608618
0.08723	-0.286	0.397215	0.964164	0.709935	0.698476	0.426921	0.357915	0.358243
-0.1144	-0.79159	-0.65939	1.47321	1.74405	1.62774	-0.33119	-0.57053	-0.19639
0.549344	0.54747	0.469552	0.530387	0.208546	0.117061	0.377674	0.475059	0.497114
0.52191	1.18357	-1.08929	0.813304	0.443015	0.945088	-1.07102	-1.47822	0.091771
0.471414	0.392181	0.162938	0.484315	0.169871	0.557496	0.179927	0.061777	0.125713
0.334538	0.675863	-0.4092	0.772597	-0.01406	0.969272	0.156344	-0.4419	0.134985
-0.44612	0.495679	0.083257	-0.18729	-1.05526	-0.39395	1.21823	1.28309	0.342398
0.108874	1.46741	0.374841	-0.90829	-0.94922	-1.24937	1.33596	1.30614	0.631107
0.61055	2.63726	-0.29817	-0.38911	-0.70985	-0.56693	-0.44052	-0.47818	-0.40202
0.183539	-0.03328	-0.09844	0.436038	0.800363	0.172285	0.349187	-0.05759	-0.23696
0.447596	0.592282	-0.29479	0.712307	0.59998	1.06397	-0.47954	-0.30354	-0.05181
0.405911	0.226441	-0.10622	-1.41319	0.740826	0.6137	0.410445	0.210637	0.695096
0.472236	0.604643	0.54261	0.251508	0.317594	0.26065	-1.62384	0.590922	0.46348
0.188991	0.70197	0.156418	-0.0395	0.41207	0.474258	0.236777	0.28639	0.174985
0.146647	0.291857	0.19351	0.259202	0.542249	0.376508	0.195026	0.195449	-0.04615
0.411311	0.593364	-0.10633	0.59697	0.579906	0.632046	0.024537	-0.0843	0.04585

Intensity 9 (Sample type D)	Intensity 11 (Sample type D)	Intensity 3 (Sample type D)	C: Modifications	C: Raw file	C: Reverse	C: Potential contaminant	C: Student's T-test Significant A_BA_vs_B	C: Student's T-test significant
0.096212	-2.5662	1.05472	Unmodifie	Nsco_20190925_Pulldowns_PH_B3_lysC				
-1.79675	-2.22881	0.900115	Unmodifie	Nsco_20190925_Pulldowns_PH_A3_lysC				
0.337001	0.366944	0.26185	Unmodifie	Nsco_20190925_Pulldowns_PH_D3_lysC				
0.645277	-1.00536	-1.69768	Unmodifie	Nsco_20190925_Pulldowns_PH_D3_lysC				
0.425729	-1.81478	-1.21569	Oxidation (	Nsco_20190925_Pulldowns_PH_C			+	A_B
0.352104	-2.06179	-2.00441	Unmodifie	Nsco_20190925_Pulldowns_PH_B			+	A_B
-0.74506	-0.67711	-0.75933	Oxidation (	Nsco_20190925_Pulldowns_PH_B			+	A_B
-1.22236	-2.77143	0.221581	Unmodifie	Nsco_20190925_Pulldowns_PH_A2_lysC				
-0.06743	-1.37136	1.07866	Unmodifie	Nsco_20190925_Pulldowns_PH_B3_lysC				
0.160995	-3.13234	0.365711	Unmodifie	Nsco_20190925_Pulldowns_PH_B3_lysC				
-0.49229	-2.69043	1.00428	Oxidation (	Nsco_20190925_Pulldowns_PH_A2_lysC				
-0.02584	-2.25907	0.944887	Unmodifie	Nsco_20190925_Pulldowns_PH_C1_lysC				
-0.73163	-0.58618	-0.79965	Arg_GlcNA	Nsco_20190925_Pulldowns_PH_C			+	A_B
-0.67082	-0.45903	1.16681	Unmodifie	Nsco_20190925_Pulldowns_PH_A2_lysC				
0.413307	-2.9496	1.02115	Unmodifie	Nsco_20190925_Pulldowns_PH_A1_lysC				
-0.30628	-2.71974	0.739576	Unmodifie	Nsco_20190925_Pulldowns_PH_B3_lysC				
-1.54366	-1.65986	1.41989	Unmodifie	Nsco_20190925_Pulldowns_PH_A3_lysC				
0.496694	-2.5324	0.155905	Unmodifie	Nsco_20190925_Pulldowns_PH_D			+	A_B
0.169204	-3.11864	0.357078	Oxidation (	Nsco_20190925_Pulldowns_PH_C3_lysC				
0.330312	-3.12363	0.63902	Unmodifie	Nsco_20190925_Pulldowns_PH_C1_lysC				
-0.11691	-3.025	0.448553	Oxidation (	Nsco_20190925_Pulldowns_PH_B3_lysC				

C: Student's T-test Significant C_DC_vs_D	C: Student's T-test significant _	N: Mass	N: Mass Fractional Part	N: Retention time	N: Calibrated retention time	N: PEP	N: Score	N: Delta score
		2914.55	0.552754	53.433	54.114	5.60E-147	187.04	177.09
		1119.61	0.60768	37.401	38.598	3.65E-35	166.07	145.23
		2546.22	0.221686	73.826	74.063	1.48E-100	175.77	169.35
		3460.68	0.675525	66.106	66.61	5.29E-74	148.26	142.25
		3476.67	0.670439	56.769	57.27	1.02E-24	97.049	93.187
		3875.85	0.845837	56.933	57.499	7.22E-13	74.581	70.124
		3891.84	0.840752	55.366	55.353	1.15E-21	96.913	87.962
		1971.02	0.020759	36.786	37.912	2.31E-145	215.93	199.62
		4191.18	0.178663	57.881	58.217	1.52E-66	132.48	123.45
		2253.23	0.230358	53.839	54.12	0	304.91	289.7
		2269.23	0.225272	49.824	50.265	3.83E-135	205	191.83
		1095.68	0.676428	45.359	46.31	1.87E-11	119.68	100.59
+	C_D	1298.76	0.755801	27.767	29.229	0.005964	96.342	60.021
		1352.81	0.813984	25.663	26.255	1.99E-11	133.88	111.24
		2238.17	0.168469	60.65	60.753	0	300.18	281.77
		2678.44	0.443187	58.67	59.157	5.96E-87	164.69	160.42
		3339.77	0.765584	62.232	62.907	6.92E-16	92.551	83.787
		1509.86	0.858885	67.22	67.598	4.83E-153	206.46	206.46
		1525.85	0.8538	46.442	46.851	3.25E-95	206.32	181.19
		1637.95	0.953848	56.84	56.938	3.51E-143	210.14	204.87
		1653.95	0.948763	47.62	47.902	9.06E-206	228.93	228.26

N: Intensity	N: MS/MS Count	N: -Log Student's T-test p-value A_BA_vs_B	N: Student's T-test Difference A_BA_vs_B	N: Student's T-test Test statistic A_BA_vs_B	N: -Log Student's T-test p-value C_DC_vs_D	N: Student's T-test Difference C_DC_vs_D	N: Student's T-test Test statistic C_DC_vs_D	T: Sequence
7.1E+09	38	1.09166	-2.60555	-2.32185	0.000208	-0.00277	-0.00064	AFWEKHSD
3.13E+09	23	0.405911	1.32241	0.95708	0.541662	5.42557	1.22641	AQLKAFWE
2.24E+11	321	0.074476	-0.0325	-0.21208	0.409891	-1.00239	-0.96511	DPPFDTEFI
1.68E+11	102	0.891021	-9.30507	-1.91151	0.879536	9.22487	1.88858	EGDPNLGV
5.34E+09	41	1.38714	-7.68774	-2.97336	0.931999	6.76424	1.99374	EGDPNLGV
6.08E+10	45	1.54802	-4.0895	-3.3597	0.952472	9.13625	2.03509	EGDPNLGV
1.88E+08	7	3.18923	-8.07858	-9.6431	-4.34E-08	0	0	EGDPNLGV
1.94E+11	237	0.859616	1.21361	1.84893	0.922146	8.75061	1.9739	GTLIVNKPC
3.99E+09	18	0.312161	-2.72907	-0.76418	0.340172	-3.61074	-0.82268	GTLIVNKPC
5.38E+11	1027	0.15303	-0.12453	-0.40969	0.365041	1.99964	0.873948	HSDIILKPLD
1.12E+11	440	0.355878	-0.87025	-0.85512	0.244898	1.56643	0.619742	HSDIILKPLD
1.51E+11	402	0.713009	3.39544	1.5606	0.630794	8.02888	1.40038	IARQIGPTLI
1.11E+09	5	1.76577	8.29843	3.92685	2.77866	8.84421	7.53099	IARQIGPTLI
1.55E+09	8	0.816308	6.42297	1.76319	0.319253	-1.88933	-0.77908	IARQIGPTLI
4.65E+11	607	1.05712	-0.68519	-2.24969	0.157201	0.792708	0.419643	LFTAWFSDI
1.36E+10	33	0.816615	-2.15352	-1.76379	0.178141	1.9164	0.468944	LFTAWFSDI
1.18E+09	13	0.097842	0.838336	0.273117	0.434967	4.44376	1.01547	LFTAWFSDI
1.44E+11	207	2.42535	0.756914	6.05473	0.134487	1.2559	0.364857	LGIVMDPIA
8.38E+09	100	0.100128	0.214022	0.278974	0.413058	3.5116	0.971491	LGIVMDPIA
3.64E+11	596	0.91798	-0.43178	-1.96553	0.276805	1.97614	0.689042	LGIVMDPIA
1.38E+11	353	0.65337	-0.88987	-1.44435	0.343436	2.61851	0.829438	LGIVMDPIA

T: Proteins	T: Gene Names	T: Protein Names	T: Identificat ion type 1	T: Identificat ion type 2	T: Identificat ion type 3	T: Identificat ion type 4	T: Identificat ion type 5	T: Identificat ion type 6
sp|P04425	gshB	Glutathion	By MS/MS	By MS/MS	By MS/MS	By MS/MS	By MS/MS	By MS/MS
sp|P04425	gshB	Glutathion	By MS/MS	By MS/MS	By MS/MS	By MS/MS	By MS/MS	By MS/MS
sp|P04425	gshB	Glutathion	By MS/MS	By MS/MS	By MS/MS	By MS/MS	By MS/MS	By MS/MS
sp|P04425	gshB	Glutathion	By MS/MS			By MS/MS	By MS/MS	By MS/MS
sp|P04425	gshB	Glutathion	By MS/MS			By MS/MS	By MS/MS	By MS/MS
sp|P04425	gshB	Glutathion	By MS/MS	By matching		By MS/MS	By MS/MS	By MS/MS
sp|P04425	gshB	Glutathione synthetase				By MS/MS	By MS/MS	By MS/MS
sp|P04425	gshB	Glutathion	By MS/MS	By MS/MS	By MS/MS	By MS/MS	By MS/MS	By MS/MS
sp|P04425	gshB	Glutathion	By MS/MS	By MS/MS	By MS/MS	By MS/MS	By MS/MS	By MS/MS
sp|P04425	gshB	Glutathion	By MS/MS	By MS/MS	By MS/MS	By MS/MS	By MS/MS	By MS/MS
sp|P04425	gshB	Glutathion	By MS/MS	By MS/MS	By MS/MS	By MS/MS	By MS/MS	By MS/MS
sp|P04425	gshB	Glutathion	By MS/MS	By MS/MS	By MS/MS	By MS/MS	By MS/MS	By MS/MS
sp|P04425	gshB	Glutathion	By matchin	By MS/MS				
sp|P04425	gshB	Glutathion	By MS/MS	By MS/MS	By MS/MS			
sp|P04425	gshB	Glutathion	By MS/MS	By MS/MS	By MS/MS	By MS/MS	By MS/MS	By MS/MS
sp|P04425	gshB	Glutathion	By MS/MS	By MS/MS	By MS/MS	By MS/MS	By MS/MS	By MS/MS
sp|P04425	gshB	Glutathion	By MS/MS	By MS/MS	By MS/MS		By matchin	By matchin
sp|P04425	gshB	Glutathion	By MS/MS	By MS/MS	By MS/MS	By MS/MS	By MS/MS	By MS/MS
sp|P04425	gshB	Glutathion	By MS/MS	By MS/MS	By MS/MS	By MS/MS	By MS/MS	By MS/MS
sp|P04425	gshB	Glutathion	By MS/MS	By MS/MS	By MS/MS	By MS/MS	By MS/MS	By MS/MS
sp|P04425	gshB	Glutathion	By MS/MS	By MS/MS	By MS/MS	By MS/MS	By MS/MS	By MS/MS

T: Identification type 7	T: Identification type 8	T: Identification type 9	T: Identification type 10	T: Identification type 11	T: Identification type 12	M: Protein group IDs
By MS/MS	By MS/MS	By MS/MS	By MS/MS		By MS/MS	869;843
By matchin	By MS/MS		By MS/MS		By MS/MS	869;843
By MS/MS	By MS/MS	By MS/MS	By MS/MS	By MS/MS	By MS/MS	869;843
By MS/MS	By MS/MS	By MS/MS	By MS/MS	By MS/MS	By MS/MS	869;843
By MS/MS	By MS/MS	By MS/MS	By MS/MS		By MS/MS	869;843
By MS/MS	By MS/MS	By MS/MS	By MS/MS		By MS/MS	869;843
						869;843
By MS/MS	By MS/MS	By MS/MS	By MS/MS		By MS/MS	869;843
		By MS/MS	By MS/MS			869;843
By MS/MS	By MS/MS	By MS/MS	By MS/MS	By MS/MS	By MS/MS	869;843
By MS/MS	By MS/MS	By MS/MS	By MS/MS	By MS/MS	By MS/MS	869;843
By MS/MS	By MS/MS	By MS/MS	By MS/MS	By matchin	By MS/MS	869
By MS/MS	By MS/MS		By matching		By MS/MS	869
						869
By MS/MS	By MS/MS	By MS/MS	By MS/MS	By MS/MS	By MS/MS	869;843
By MS/MS	By MS/MS	By MS/MS	By MS/MS		By MS/MS	869;843
By MS/MS	By MS/MS		By MS/MS		By MS/MS	869;843
By MS/MS	By MS/MS	By MS/MS	By MS/MS	By MS/MS	By MS/MS	869;843
By MS/MS	By MS/MS	By MS/MS	By MS/MS		By MS/MS	869;843
By MS/MS	By MS/MS	By MS/MS	By MS/MS	By MS/MS	By MS/MS	869;843
By MS/MS	By MS/MS	By MS/MS	By MS/MS	By MS/MS	By MS/MS	869;843

Proteins	Positions within proteins	Leading proteins	Protein	Protein names	Gene names	Fasta headers	Localization prob	Score diff
sp|P04425	256;256	sp|P04425	sp|P04425	Glutathion	gshB		1	70.0564

PEP	Score	Delta score	Score for localizatio n	Number of Arg_GlcNA c	Amino acid	Sequence window	Modificati on window	Peptide window coverage
0.005964	100.09	49.937	70.056	1	R	RGEPRPLTE	X;X;X;X;X;X	XXXXXXXXX

Arg_GlcNAc Probabilities	Arg_GlcNAc Score diffs	Position in peptide	Charge	Mass error [ppm]	Identification type 1 (Sample type A)	Identification type 12 (Sample type A)	Identification type 2 (Sample type A)	Identification type 4 (Sample type B)
IAR(1)QIGP	IAR(70.06)(	3	3	-0.30548	By matchin	By MS/MS	By MS/MS	

Identification type 5 (Sample type B)	Identification type 6 (Sample type B)	Identification type 7 (Sample type C)	Identification type 8 (Sample type C)	Identification type 10 (Sample type C)	Identification type 3 (Sample type D)	Identification type 11 (Sample type D)	Identification type 9 (Sample type D)	Intensity
		By MS/MS	By MS/MS	By matching				1.11E+09

Intensity___1	Intensity___2	Intensity___3	Ratio mod/base	Intensity 1	Intensity 10	Intensity 11	Intensity 12	Intensity 2
1.11E+09	0	0	0.007363	2483300	12570000	0	4429600	1.45E+08

Intensity 3	Intensity 4	Intensity 5	Intensity 6	Intensity 7	Intensity 8	Intensity 9
0	0	0	0	1.05E+08	1.01E+08	0

Ratio mod/base 1 (Sample type A)	Ratio mod/base 12 (Sample type A)	Ratio mod/base 2 (Sample type A)	Ratio mod/base 4 (sample type B)	Ratio mod/base 5 (sample type B)	Ratio mod/base 6 (sample type B)	Ratio mod/base 7 (sample type C)
0.03464	0.007472	0.047244	0	0	0	0.001918

Sample type A Occupancy (GshB-FLAG-His , Citrobacter strain expressing wild-type, endogenous NleB)	2.98%
Sample type C Occupancy (His-GshB-His incubated in vitro with wild-type GST-NleB-His)	0.36%

Ratio mod/base 8 (sample type C)	Ratio mod/base 10 (sample type C)	Ratio mod/base 3 (sample type D)	Ratio mod/base 11 (sample type D)	Ratio mod/base 9 (sample type D)	Intensity 1___1	Intensity 1___2	Intensity 1___3	Intensity 10___1
0.001419	0.007539	0	0	0	2483300	0	0	12570000

Intensity 10___2	Intensity 10___3	Intensity 11___1	Intensity 11___2	Intensity 11___3	Intensity 12___1	Intensity 12___2	Intensity 12___3	Intensity 2___1
0	0	0	0	0	4429600	0	0	1.45E+08

Intensity 2___2	Intensity 2___3	Intensity 3___1	Intensity 3___2	Intensity 3___3	Intensity 4___1	Intensity 4___2	Intensity 4___3	Intensity 5___1
0	0	0	0	0	0	0	0	0

Intensity 5___2	Intensity 5___3	Intensity 6___1	Intensity 6___2	Intensity 6___3	Intensity 7___1	Intensity 7___2	Intensity 7___3	Intensity 8___1
0	0	0	0	0	1.05E+08	0	0	1.01E+08

Intensity 8___2	Intensity 8___3	Intensity 9___1	Intensity 9___2	Intensity 9___3	id	Protein group IDs	Positions	Position
0	0	0	0	0	21	869	256	256

Peptide IDs	Mod. peptide IDs	Evidence IDs	MS/MS IDs	Best localization evidence ID	Best localization MS/MS ID	Best localization raw file	Best localization scan number	Best score evidence ID
1434	1483	12052;120	9739;9740	12056	9743	Nsco_2019	5088	12052

Best score MS/MS ID	Best score raw file	Best score scan number	Best PEP evidence ID	Best PEP MS/MS ID	Best PEP raw file	Best PEP scan number
9739	Nsco_2019	4866	12054	9741	Nsco_2019	5401

Table S2. List of GshB peptides analyzed using EthcD mass spectrometry.
