# Supplementary figures and images for: An intra-bacterial activity for a T3SS effector
Source: Sci Rep. 2020 Jan 23;10:1073. doi: 10.1038/s41598-020-58062-y (PMC6978387; doi:10.1038/s41598-020-58062-y)

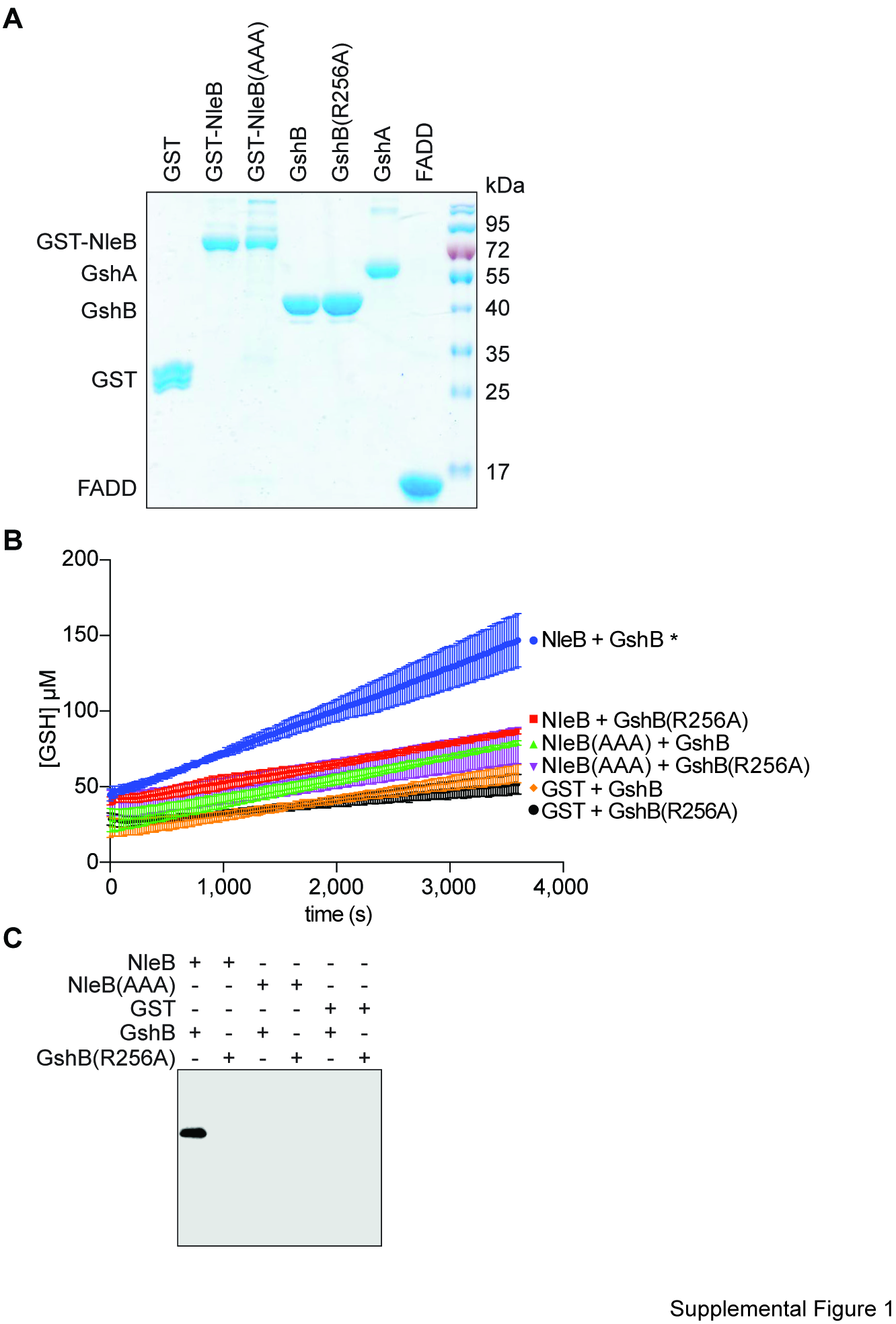

Supplement: Supplementary file 2 — Supporting Information2. [file 41598_2020_58062_MOESM2_ESM.tif]

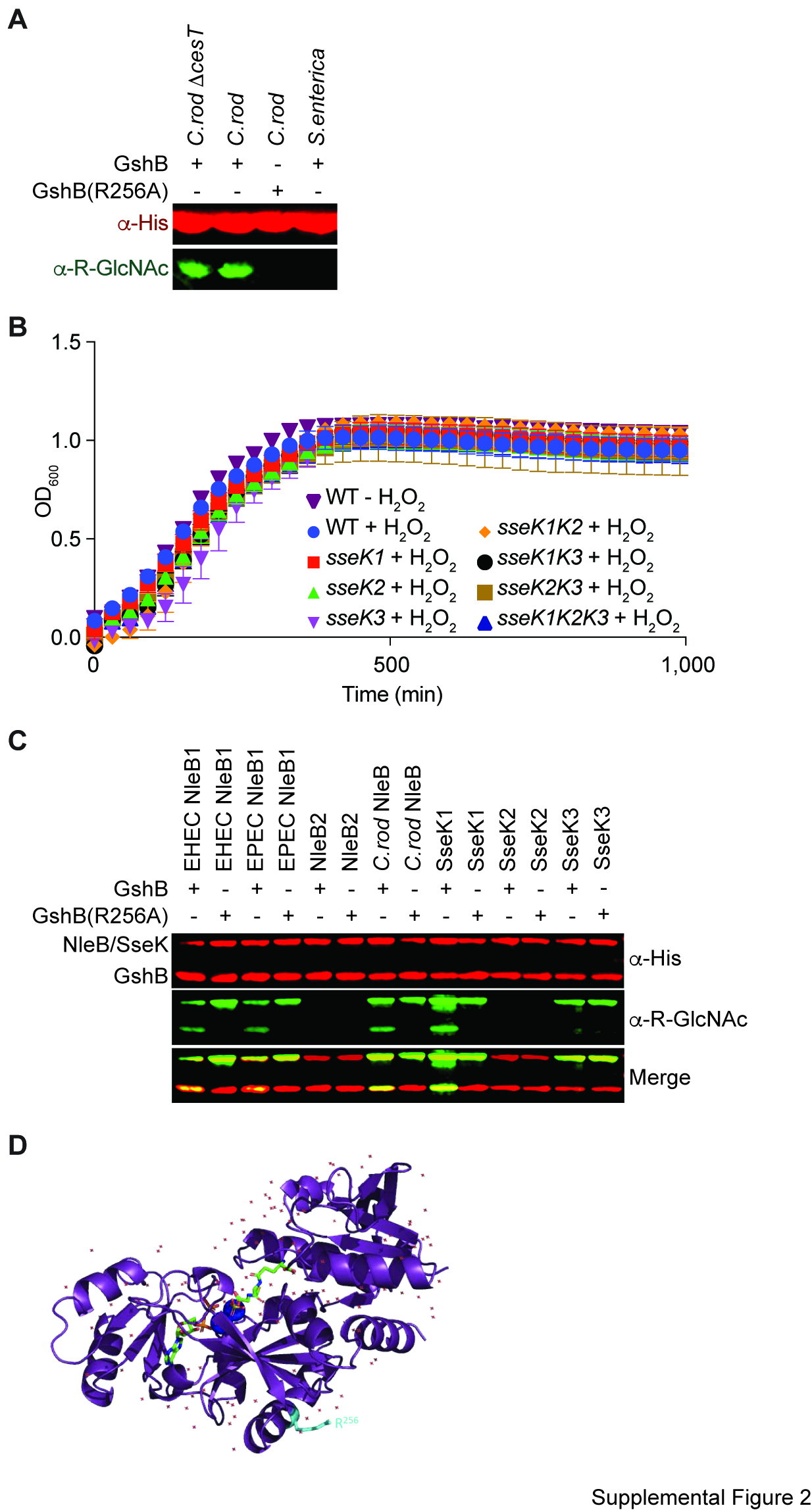

Supplement: Supplementary file 3 — Supporting Information3. [file 41598_2020_58062_MOESM3_ESM.tif]

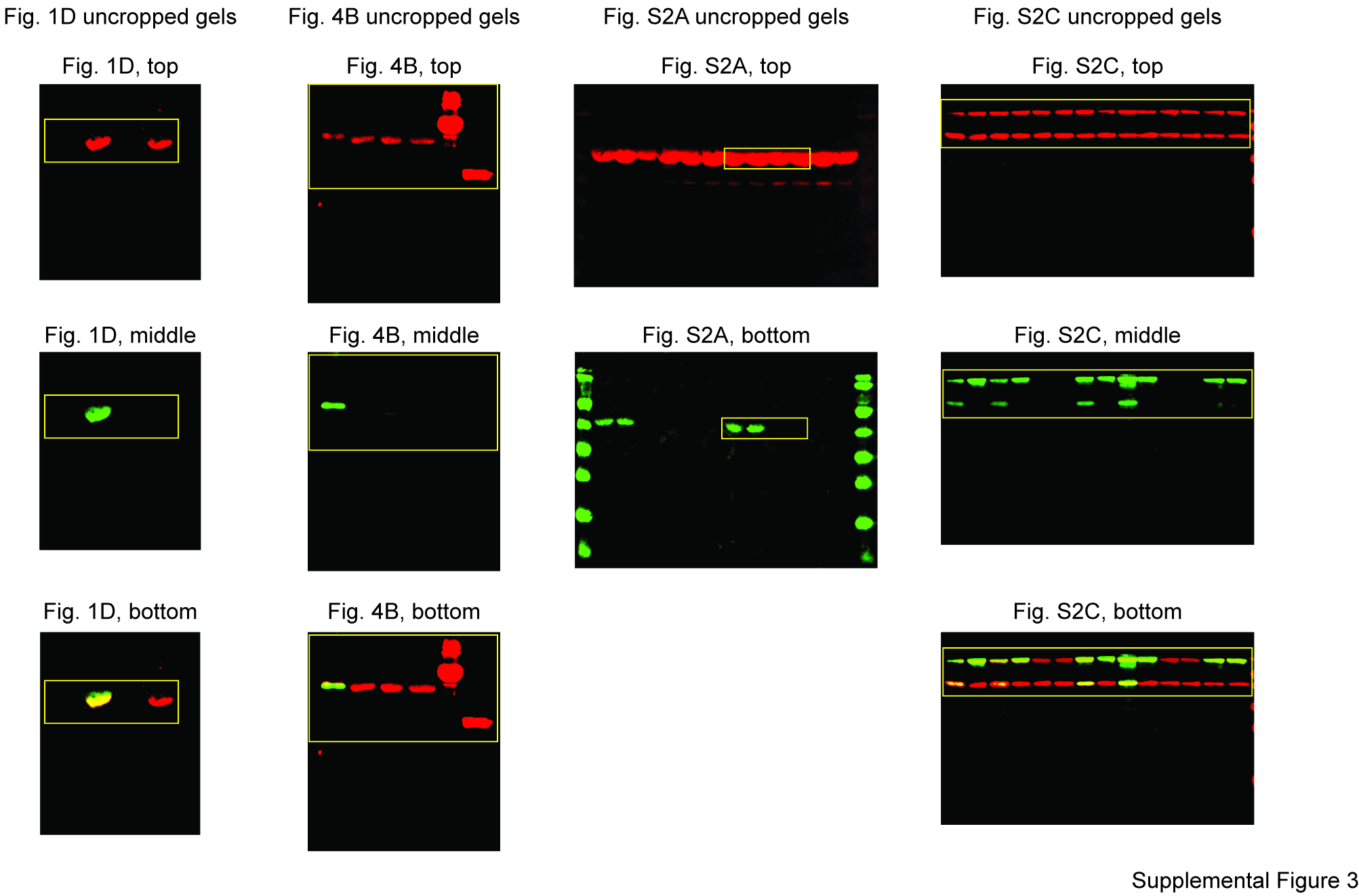

Supplement: Supplementary file 4 — Supporting Information4. [file 41598_2020_58062_MOESM4_ESM.tif]
