# Supplementary material for: An intra-bacterial activity for a T3SS effector
Source: Sci Rep. 2020 Jan 23;10:1073. doi: 10.1038/s41598-020-58062-y (PMC6978387; doi:10.1038/s41598-020-58062-y)
Supplement: Supplementary file 6 — Supporting Information6. [file 41598_2020_58062_MOESM6_ESM.pdf]

Proteins	Positions within proteins	Leading proteins	Protein	Protein names
B7UHZ4	256	B7UHZ4	B7UHZ4	Glutathione synthetase
Q13158	117	Q13158	Q13158	FAS-associated death domain protein
B7UK20	56	B7UK20	B7UK20	30S ribosomal protein S4
B7UPA2	43	B7UPA2	B7UPA2	Pseudouridine synthase
B7UPA2	48	B7UPA2	B7UPA2	Pseudouridine synthase
B7UQM2	76	B7UQM2	B7UQM2	Peptidyl-prolyl cis-trans isomerase
B7UID2	159	B7UID2	B7UID2	Ribosomal RNA small subunit methyltransferase
B7UQ78	222	B7UQ78	B7UQ78	Flagellar brake protein YcgR
B7UQ78	226	B7UQ78	B7UQ78	Flagellar brake protein YcgR

Gene names	Localization prob	Score diff	PEP	Score	Delta score	Score for localization
gshB	1	79.0365	8.01E-24	204.01	153.73	79.036
FADD	1	162.722	3.45E-12	170.26	123.38	162.72
rpsD	1	129.006	2.53E-06	129.01	101.04	129.01
rluC	0.5	0	0.00134749	126.03	81.386	115.41
rluC	0.5	0	0.00134749	126.03	81.386	115.41
fklB	1	106.656	6.58E-06	106.66	69.512	106.66
rsmH	1	105.204	0.00247799	105.2	92.707	105.2
ycgR	0.5	0	1.41E-06	100.43	52.21	100.43
ycgR	0.5	0	1.41E-06	100.43	52.21	100.43

Amino acid	Sequence window	Modification window	Peptide window coverage	Arg_GlcNAc Probabilities	Arg_GlcNAc Score diffs	Position in peptide
R	RGEPRPLTES	X;X;X;X;X;X;X;	XXXXXXXXXXX	IAR(1)QIGPT	IAR(79.04)QI	3
R	NVICDNVGKI	X;X;X;X;X;X;X;	XXXXXXXXXXX	LAR(1)QLKVS	LAR(162.72)(	3
R	HGARKPRLSI	X;X;X;X;X;X;X;	XXXXXXXPPPI	LSDYGVQLR(	LSDYGVQLR(	9
R	TQLKGVPKSN	X;X;X;X;X;X;X;	XXXXXXXXXXX	ILR(0.5)KGEV	ILR(0)KGEVR	3
R	VPKSMIYRILF	X;X;X;X;X;X;X;	XXXXXXXXXXX	ILR(0.5)KGEV	ILR(0)KGEVR	8
R	ALREIHERAD	X;X;X;X;X;X;X;	XXXXXXXXXXX	QR(1)FQAM/	QR(106.66)F	2
R	EEADIAWVLK	X;X;X;X;X;X;X;	XXXXXXXXXXI	TYGEER(1)F/	TYGEER(105	6
R	PRLSFRFLNV	X;X;X;X;X;X;X;	XXXXXXPPPPI	FLNVSPTVER	FLNVSPTVER	10
R	FRFLNVSPTV	X;X;X;X;X;X;X;	XXXXXXXXXXX	FLNVSPTVER	FLNVSPTVER	14

Charge	Mass error [ppm]	Identification type 1	Identification type 2	Intensity	Intensity___1	Ratio mod/base
3	-0.21513	By MS/MS	By MS/MS	262180000	262180000	NaN
2	0.21524	By matching	By MS/MS	37690000	37690000	NaN
3	-0.048721	By MS/MS	By MS/MS	10343000	10343000	2.0275
3	0.10753	By MS/MS	By MS/MS	35524000	35524000	NaN
3	0.10753	By MS/MS	By MS/MS	35524000	35524000	NaN
3	-0.13725	By MS/MS	By MS/MS	11107000	11107000	NaN
2	0.13306	By matching	By MS/MS	4837200	4837200	NaN
3	-0.060312		By MS/MS	3228400	3228400	NaN
3	-0.060312		By MS/MS	3228400	3228400	NaN

Intensity 1	Intensity 2	Ratio mod/base 1	Ratio mod/base 2	Ratio mod/base 3	Intensity 1___1	Intensity 2___1
42409000	48442000	NaN	NaN	NaN	42409000	48442000
1267800	2286000	NaN	NaN	NaN	1267800	2286000
4196100	6147100	12.468	NaN	0	4196100	6147100
13189000	22335000	NaN	NaN	NaN	13189000	22335000
13189000	22335000	NaN	NaN	NaN	13189000	22335000
4720800	4261100	NaN	NaN	NaN	4720800	4261100
963850	3873300	NaN	NaN	NaN	963850	3873300
0	3228400	NaN	NaN	NaN	0	3228400
0	3228400	NaN	NaN	NaN	0	3228400

id	Protein group IDs	Positions	Position	Peptide IDs	Mod. peptide IDs	Evidence IDs
7	537	256	256	4132;4133	4277;4278	11690;11691
40	2786	117	117	4819	4973	13254;13255
12	744	56	56	5201	5364	14141;14142
21	1127	43	43	4322	4467	12104;12105
22	1127	48	48	4322	4467	12104;12105
26	1238	76	76	6189	6410	16515;16516
8	566	159	159	10891	11277	29303;29304
24	1204	222	222	1383	1408	3089
25	1204	226	226	1383	1408	3089

MS/MS IDs	Best localization evidence ID	Best localization MS/MS ID	Best localization raw file	Best localization scan number	Best score evidence ID	Best score MS/MS ID
10089;10090	11694	10092	Nsco_20181[illegible]	7390	11692	10091
11359;11360	13257	11363	Nsco_20181[illegible]	6034	13255	11361
12068;12069	14142	12069	Nsco_20181[illegible]	13252	14142	12069
10418;10419	12105	10419	Nsco_20181[illegible]	3983	12104	10418
10418;10419	12105	10419	Nsco_20181[illegible]	3983	12104	10418
14012;14013	16518	14015	Nsco_20181[illegible]	10286	16518	14015
24897	29303	24897	Nsco_20181[illegible]	7427	29303	24897
2505	3089	2505	Nsco_20181[illegible]	20271	3089	2505
2505	3089	2505	Nsco_20181[illegible]	20271	3089	2505

Best score raw file	Best score scan number	Best PEP evidence ID	Best PEP MS/MS ID	Best PEP raw file	Best PEP scan number
Nsco_201812	9361	11692	10091	Nsco_201812	9361
Nsco_201812	5781	13255	11361	Nsco_201812	5781
Nsco_201812	13252	14142	12069	Nsco_201812	13252
Nsco_201812	3971	12104	10418	Nsco_201812	3971
Nsco_201812	3971	12104	10418	Nsco_201812	3971
Nsco_201812	10286	16518	14015	Nsco_201812	10286
Nsco_201812	7427	29303	24897	Nsco_201812	7427
Nsco_201812	20271	3089	2505	Nsco_201812	20271
Nsco_201812	20271	3089	2505	Nsco_201812	20271

Table S1. List of proteins containing Arg-GlcNAcylated peptide enriched from infection samples.
